# The PREvention Program for Adolescent Relationship and Emotional Development (PREPARED): a proof-of-concept study

**DOI:** 10.1186/s12889-026-26880-w

**Published:** 2026-03-16

**Authors:** Savannah L. Johnson, Justin M. Rasmussen, Madalyn Bielskis, Allison Falls, Halima Z. Hamisi, Oyombe J. Owity, Eve S. Puffer

**Affiliations:** 1 Department of Psychology and Neuroscience, Duke University, 417 Chapel Dr, Durham, NC 27708 United States of America; 2 Duke Global Health Institute, 310 Trent Dr, Durham, NC 27710 United States of America; 3 Present Address: WISER Girls Secondary School, PO Box 28–4049, Muhuru Bay, Kenya; 4 Massachusetts General Hospital, Department of Psychiatry, 55 Fruit Street, MA 02114 Boston, United States of America

**Keywords:** Adolescent mental health, Intimate partner violence, Implementation science, Global mental health, Kenya, Community based participatory research

## Abstract

**Background:**

The bi-directional link between mental health problems and intimate partner violence (IPV) is well established, yet there is a lack of prevention approaches directly targeting both during adolescence. This study conducted a mixed-methods pilot evaluation of the Prevention Program for Adolescent Relationship and Emotional Development (PREPARED), a weekly, six-session, non-specialist delivered, prevention program to promote mental health and prevent IPV. This proof-of-concept study tested implementation outcomes and measured mental health and relationship attitude and behavior changes among adolescents in rural Kenya.

**Methods:**

PREPARED was piloted with 46 adolescents aged 14–17 (*M* = 15.47, *SD* = 1.1) over six weeks. Each session was designed to last approximately two hours, totaling 12 h. Two implementation settings were tested to reach youth enrolled in secondary school (*N* = 22) and youth not enrolled in school (*N* = 24). Young adult non-specialist providers (*N* = 10) were trained to deliver the intervention. Implementation outcomes were assessed at the participant and facilitator levels through surveys, tracking, and qualitative focus group discussions (FGDs). Adolescent participants completed pre-post surveys (immediately before and following the intervention) and endline FGDs to assess changes in mental health and relationship attitudes and behaviors.

**Results:**

Participant and facilitator implementation results suggest PREPARED is feasible, acceptable, appropriate, adopted, and accessible in both school and community settings. On average, youth attended 5.5 sessions out of 6; 75% attended all 6. Providers facilitated the intervention with high fidelity (*M* = 97.8%). Tests of preliminary effectiveness revealed promising, though modest, changes in mental health outcomes and greater agreement with positive approaches to sexual relationships. We did not observe changes in disagreement with male-perpetrated violence in the overall sample. Among adolescents in the same dating relationship throughout the intervention (*N* = 20), communication in dating relationships improved and shared decision-making increased. There were no substantial changes in reported IPV victimization and perpetration immediately post-intervention.

**Conclusions:**

Our study supports the feasibility and acceptability of a novel non-specialist peer-provided mental health and IPV combined prevention intervention in a low-resource setting. The study also provides preliminary positive pre-post signals of change in mental health outcomes and some relationship-related processes, but no immediate change in IPV victimization and perpetration.

**Trial registration:**

Open Science Framework (https://doi.org/10.17605/OSF.IO/3H6XT).

**Supplementary Information:**

The online version contains supplementary material available at 10.1186/s12889-026-26880-w.

## Background

Intimate partner violence (IPV) often emerges in adolescence, with about 24% of 15–19 girls and young women experiencing sexual or physical IPV [1]. Most interventions address IPV in adults, which is often too late to prevent the deleterious outcomes of victimization and perpetration, including serious mental health problems [2–7]. Mental health problems, however, are not only adverse outcomes of IPV, but also potential drivers [8, 9]. This bidirectional relationship between mental health problems and experiences of IPV has been established in adult relationships [8–11], yet remains understudied in adolescent populations.

Despite this link, there is a lack of adolescent interventions that are designed to directly target and prevent the emergence of mental health problems *and* partner violence in low- and middle-income countries (LMICs) [12, 13]. Instead, most are distinctly designed for either mental health promotion or IPV prevention. IPV focused interventions have been tested in both high-income countries (HICs) and LMICs, yet existing evidence is almost exclusively drawn from the United States and sub-Saharan Africa (SSA) [7, 13, 14]. Implemented primarily in schools, IPV prevention programs often take a universal prevention approach (i.e., no criteria for participation, aimed to reduce risks and enhance health) [7]. In HICs, interventions typically address IPV victimization and perpetration among boys and girls, while in LMICs, most programs focus exclusively on victimization among girls [7]. Evidence strongly supports the inclusion of both sexes in violence prevention initiatives, which is recognized as a critical component of successful strategies to reduce violence against women and girls [15]. In SSA, where the burden of HIV is high, many IPV prevention programs are combined with HIV prevention and sexual reproductive health promotion [16]. Although mechanistic gaps remain in understanding violence prevention in adolescence, existing programs in LMICs emphasize transforming gender-related attitudes and relationship norms to promote behavior change, alongside key implementation strategies such as peer-led facilitation, developmentally appropriate content, and creating safe environments where young people can discuss sensitive topics [17].

Mental illness, like IPV, can emerge during adolescence as nearly half of all mental illnesses develop by the age of 14 [18]. Like IPV prevention efforts, most of the existing evidence is drawn from HICs [19–21]. Schools are the most common setting for adolescent mental health promotion, but interventions have also been implemented in community settings and through digital platforms [19]. Cognitive behavioral therapy (CBT) is the primary theoretical orientation of most of these interventions and has been shown to be effective in reducing depressive and anxiety symptoms in school-based, group delivery models in both HICs and LMICs [19, 20, 22]. In LMICs specifically, there is also evidence supporting non-specialist delivery of mental health interventions [22]. Task sharing, the training of non-specialist providers to deliver interventions, is a common and effective implementation strategy in LMICs where mental health services are often limited [23–25].

Given the intersections between mental health problems and IPV, as well as the shared implementation settings and modes of delivery, there are opportunities for combined approaches and calls to develop and test them [5]. We address this gap by developing and piloting a combined mental health promotion and IPV prevention intervention for adolescents in rural southwestern Kenya. A qualitative formative study in this community revealed that dating relationships began at an early age and were characterized by co-occurring mental health issues and IPV, driven by unmet financial and emotional needs within families, and conducted secretly without parental knowledge or supervision. Youth commonly engaged in both same-age dating relationships and age-disparate relationships involving sexual transactions with older partners. Factors contributing to IPV included the normalization of violence as a conflict resolution strategy, unclear relationship expectations, and perceived or actual unfaithfulness [26]. Given the lack of specialized mental health services and the high number of adolescents who are not enrolled in secondary school (i.e., due to economic barriers and lack of school fees) in this setting, the intervention was designed for non-specialist delivery in a school-based and a community-based setting. In this proof-of-concept study, we first aimed to conduct a preliminary test of implementation outcomes (acceptability, adoption, appropriateness, feasibility, and reach/accessibility), and second, aimed to measure preliminary changes in adolescents' mental health and dating relationship attitudes and behaviors.

## Methods

This proof-of-concept used mixed-methods to assess both implementation outcomes and preliminary effectiveness. For aim one, implementation outcomes in a school-based and a community-based setting were evaluated through surveys, detailed tracking, and qualitative focus group discussions (FGDs). For aim two, preliminary effectiveness on mental health, sexual health, and relationship attitudes and behaviors was measured using a pre-post single group design and endline FGDs.

### Setting

This study was conducted in Muhuru Bay, a rural fishing community located on the coast of Lake Victoria in Migori County, in southwestern Kenya. The region is characterized by longstanding educational and economic gender inequity [27–29]. The public health burden in this area is among the most severe in Kenya. At the county level, HIV prevalence is estimated at 14.7%—well above the national average—with rates in lakeside fishing communities reaching as high as 32% [30, 31]. Further, the lifetime prevalence of IPV in this region is high; in a neighboring Migori County community the prevalence is estimated at 60% among women over the age of 15 [32, 33].

These overlapping epidemics of HIV and IPV create particularly challenging circumstances for the region's adolescent population. Evidence from this community suggests early sexual debut and the existence of early romantic or sexual relationships among adolescents that intersect with psychosocial stressors and the emergence of adolescent IPV [26, 34]. The significant psychosocial needs among adolescents, including depression and suicidality, are further compounded by HIV exposure, AIDS-related deaths in families, poverty, and experiences of IPV [26, 35–38]. Despite these pressing challenges, there are few to no accessible formalized mental health services and few resources to respond to relationship violence.

### Community based participatory research

This study was guided by community-based participatory research (CBPR) methods [39, 40]. CBPR, as defined by Minkler and Wallerstein [41], is a collaborative orientation to research that “equitably involves community members, researchers, and other stakeholders in the research process and recognizes the unique strengths that each bring.” We followed the steps outlined by Collins and colleagues [42] for CBPR for community involvement in psychology research. An initial partnership was established between the research team and a local organization, the Women’s Institute of Secondary Education and Research (WISER). WISER is a community organization and secondary school for girls. It served as the local host for the larger, multiphase research study. To engage other community stakeholders, the sub county commissioner hosted a community meeting that included local government officials and local community members from within each sub-location within Muhuru Bay. During the meeting, community officials and members discussed: 1) the primary challenges faced by youth in the community, 2) existing infrastructure for supporting youth, 3) the potential and challenges of research-community partnerships. From this meeting, a community advisory board (CAB) was established to represent numerous sectors (e.g., religious, education, business, government) and the perspectives of adolescents themselves. Some CAB members were nominated directly from the initial community meetings; others were identified and invited based on their existing role in the community. The CAB consisted of 13 members, including the principal investigator on this study (names and roles are included in Supplemental Material A). The CAB worked together to co-create research questions related to adolescent mental health and relationship behaviors, complete qualitative formative work [26], design the study intervention, and pilot the intervention.

### Intervention

The PREvention Program for Adolescent Relationship and Emotional Development (PREPARED) is a structured, six-session, group-based combined mental health promotion and IPV prevention program. The process of development was informed by the implementation science Transcreation Framework [43] for intervention development to address health disparities, which is compatible with the CBPR approach of the overall study. The Transcreation Framework includes seven steps: 1) identify community infrastructure and engage partners; 2) specify theory; 3) identify inputs for new program; 4) design intervention prototype; 5) design study, methods, and measures for community setting; 6) build community capacity for delivery; and 7) deliver intervention and evaluate implementation processes.

Two intervention development workshops took place with the CAB. The first workshop was focused on developing a theory of change and an intervention outline. Based on the formative findings from the community [29] and participatory mind-mapping (photos included in Supplemental Materials B), the team created a hypothesized theory of change (displayed in Fig. 2). The theory of change includes individual processes (improved emotion regulation, increased healthy coping, and increased knowledge of sexual health) as well as relationship-related changes (greater acceptance of equitable power dynamics, improved communication skills, and greater engagement with natural supports). A six-session outline (Table 1) was developed to align with the hypothesized theory of change. The second workshop focused on intervention implementation planning. The CAB voted on whether the intervention should be designed for younger (10–13-year-olds) or older adolescents (14–17-year-olds). With the goal of reaching more youth with experience in dating relationships, they decided to focus on older adolescents for this preliminary study with the understanding that some in that age group are likely already experiencing violence. The CAB determined the content combines didactics, discussion, participatory demonstrations, games, worksheets, and skits. They also recommended structuring sessions with two components: mixed-sex segments for general teaching and sex-segregated breakout groups where participants could more comfortably discuss sensitive topics like mental health challenges, sexual behaviors, and violence. Mixed-sex sessions included all adolescents together, while sex-segregated sessions separated participants by sex (all males present, or all females present). The sex-segregated groups were implemented at the same time, and one group would go to another nearby location (e.g., another room or outside).

**Table 1:** PREPARED session outline

General Component	Session	Session Title	Specific Topics
Mental Health Content	1	I am prepared to notice my feelings	• Introduction to PREPARED • Emotion Identification • Relaxation/Breathing
	2	I am prepared to cope	• Substance Use Prevention • Emotion Regulations Skills
Relationship Content	3	I am prepared for healthy relationships	• Healthy Relationship Education • Relationship Communication Skills
	4	I am prepared for safe relationships	• Gender Equity/Human Dignity Rights • Sexual Health Education • Consent Training
Combined Content	5	I am prepared to work together	• Problem Solving • Conflict Resolution
	6	I am prepared to seek support	• Individual and relationship goal setting • Support Planning • Caregiver-Youth Game

Based on these workshops and the intervention outline, intervention sessions were drafted one at a time by the lead author (SJ). As each session was drafted, the CAB met, reviewed each session in detail, and provided suggested revisions. Revisions were made and feedback was incorporated in the draft of the subsequent sessions. This iterative process continued over the course of two months until there was a complete, 6-session intervention prototype.

### Participants

Adolescents between the ages of 14 and 17 who resided within the study community were eligible for the study regardless of whether they reported being in a dating relationship. Adolescents in the school-based group were recruited from a centrally located, public, mixed-sex, day school that partnered with the study. The school principal and counseling teacher invited 24 adolescents enrolled in Form 2 (i.e., second year of secondary school) and tried to ensure sex and community region representation. Two adolescents were excluded because one could not be located at the time for consent and one did not complete their survey. The community-based group, for adolescents not enrolled in school, was recruited by CAB members to equally represent the study community’s ten sublocations. Thirty-one adolescents were recruited from the community, but seven were excluded for not being located at the time of consent or opting out of the study. The final sample consisted of 46 adolescents: 22 school-based and 24 community-based adolescents. Written parental or guardian permission and written youth assent were obtained before data collection. The ability to withdraw from the study, not answer questions, or not participate in certain aspects of the study was emphasized more than usual during the permission and assent processes given the sensitive nature of the topics. Further, parents/guardians and adolescents were made aware of the appropriate follow ups and safeguarding procedures if concerns for safety were reported.

**Fig. 1:**
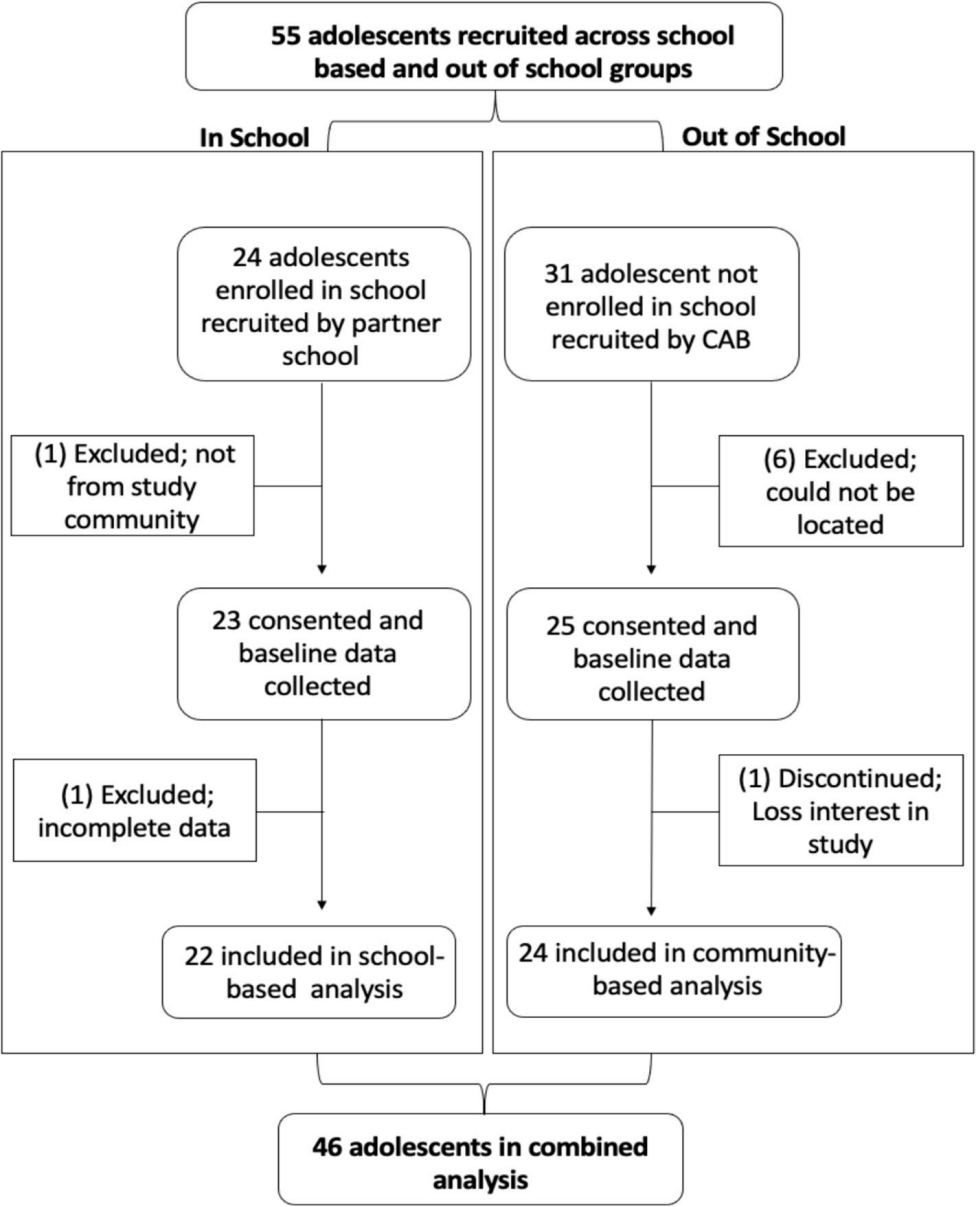
PREPARED participant flow diagram

A participant flow diagram is presented in Fig. 1.

We tested a task-shifted model of delivery by selecting and training non-specialist “near-peer” facilitators (i.e., close in age but slightly older with shared lived experiences) with no prior mental health training. Eligibility criteria for facilitators included being age 20 to 25, having language proficiency in English, Kiswahili, and Luo, and being from the study community. The CAB handed out applications in each of the community sublocations. Four CAB members and two research assistants facilitated the selection process which involved an application and in-person interview. The applications included questions about education level, previous experiences working with youth, as well as their availability. During the interviews, participants were asked to come prepared to demonstrate an activity or song that could act as an “energizer” for a group of youth. Each applicant was rated on their group facilitation and communication abilities. A total of 28 young adults applied and the five young men and five young women with the highest ratings, who also represented the community sublocations, were selected and invited to attend the facilitator training.

### Procedures

#### Facilitator training

Facilitator training took place over 12 days (~ 60 contact hours) and was led by one of the lead researchers (SJ) and local research team members (JO, HH). Training involved a combination of didactics, group discussion, role play, and practice facilitation of intervention sessions. Didactic and group discussion topics included psychoeducation on adolescent mental health, trauma-informed approaches to discussing mental health and violence, health relationship principles, communication skills, etc. Mental health promotion training included basic supportive counseling skills and active listening techniques. Facilitators were trained to manage mild psychological distress within sessions through supportive discussion and normalization. A clear referral protocol was established whereby facilitators would refer participants to study PI for follow up assessment when participants exhibited suicidal ideation, severe depression or anxiety symptoms, disclosed recent IPV or other violence. At the end of training, the group of 10 facilitators were split into two groups (five for the school-based implementation and five for the community-based implementation). In each group, there were two lead facilitators (one male and one female) who were selected based on their facilitation abilities during the training. The remaining facilitators in each group served as support facilitators who also facilitated, but only smaller portions of the sessions, and assisted with attendance tracking, ensuring adolescents completed worksheets, and answering questions.

#### Intervention implementation

The intervention was implemented weekly over the course of six weeks during June and July of 2023. Each session lasted approximately two hours. In the school-based setting, sessions occurred weekly at the school on Sunday afternoons when many students were already at the school studying. The community-based implementation took place in a fishing hall at one of the primary lake beaches in the community. The CAB determined that this fishing hall would be an ideal location for youth who are not enrolled in school to gather because it is not a stigmatized location, it is centrally located, and it is often unused by fisherman in the late afternoons. Sessions occurred on Wednesday afternoons. Adolescents in both groups were provided with a small snack during each session and were provided with a small token of appreciation for participation (e.g., bar of soap, bag of flour or sugar, and phone airtime).

#### Data collection

Adolescents completed pre-post surveys interviews with a trained research assistant from the community using a tablet running Qualtrics data collection software [44]. Adolescents in dating relationships completed a longer survey that included measures about relationship-specific attitudes and behaviors. Adolescents in relationships also answered IPV-related questions and entered responses on the tablets themselves given the sensitive nature of the topic. The surveys were translated and back translated from English to Kiswahili and Luo and were available in all three languages. Participants completed surveys in whichever language they were most comfortable. Survey data collection took place the week before the first session of the intervention and the week after the final session. All participants were also invited to participate in post-intervention qualitative FGDs facilitated by research assistants. There were four adolescent FGDs organized by implementation setting and sex (i.e., school-based female, school-based male, community-based female, community-based male). Participants were compensated for research-related tasks; school-based participants received calculators and community-based participants received an equivalent amount in Kenyan shillings (~ 2000 KSH). Cost for transportation to sessions was also provided for the community-based youth.

Implementation data were collected from intervention facilitators as well. They provided written informed consent for all research-related activities. Facilitators completed a post-intervention survey, self-administered on a tablet running Qualtrics. They also participated in a FGD about experiences as a facilitator. Facilitators were compensated for implementation of the intervention (1000 KSH per session) and for research-related tasks (500–1000 KSH per task). All compensation decisions were made by the CAB to ensure appropriateness for the context.

#### Ethical review

Ethics approval was obtained from the Strathmore University Institutional Scientific Ethics Review Committee (Protocol: SU-ISERC1615/23) and the Duke University Campus Institutional Review Board (Protocol: 2022–0500). The protocol was registered through the Open Science Framework on May 17, 2023 (Preregistration: 10.17605/OSF.IO/3H6XT).

### Measures

#### Demographics and adverse child experiences

For adolescents, we collected demographic information including age, sex, educational level, primary caregiver or parent, household composition, religious affiliation, relationships status, age at sexual debut, and if they have children. We also assessed adverse childhood experiences (ACEs) to understand histories of adversity upon starting the intervention. We used 21-items from the World Health Organization’s Adverse Child Experiences International Questionnaire (ACE-IQ) [45]. The selected items measure adversity related to childhood neglect and abuse, family adversity, and bullying. We used the frequency scoring scheme to create a cumulative ACE score ranging 0–10 (α = 0.71).

For facilitators, we collected age, sex, level of education, marital status, religious affiliation, previous leadership experience, and previous experiences working with youth.

#### Implementation outcomes: surveys and tracking

Implementation-related domains were conceptualized and defined based on the Implementation Outcomes Framework [46] and were assessed using the Mental Health Implementation Science Tools (mhIST) and include surveys from the provider (intervention facilitators) and the consumer (adolescent) perspectives [47]. This tool has been adapted and used in other LMICs, including another country in sub-Saharan Africa [34], and were tailored for the purpose of assessing preliminary implementation outcomes of PREPARED.

Acceptability (i.e., satisfaction) of PREPARED was assessed at the adolescent level with a six-item subscale and at the facilitator level with an eight-item subscale. Example item: “Do you feel that the skills you learned in PREPARED are useful?” To assess Adoption (i.e., uptake, willingness to participate) of PREPARED, adolescents completed a six-item subscale, and facilitators completed an eight-item subscale. Example item: “Have you used the skills you learned in PREPARED?” Appropriateness (i.e., perceived fit or relevance of intervention for the context) was assessed by adolescents with a seven-item subscale and by facilitators with a five-item subscale. Example item: “Does PREPARED fit with your cultural values?” Feasibility (i.e., extent to which PREPARED can be carried out successfully) was assessed at the facilitator level with a ten-item subscale that included items about the time, skills, and resources required for implementation. At the adolescent level, feasibility was assessed with an eight-item subscale that assessed how well the program fit within daily life. Example item: “Do you have enough time for all the activities that go into PREPARED?” Feasibility was also assessed by tracking facilitator and adolescent attendance with attendance logs. Reach/Accessibility (i.e., perceived likelihood of others seeking or accessing PREPARED) was assessed through a four-item subscale by facilitators and a six-item subscale by adolescents. Example item: “Would female youth who need emotional or relationship support seek PREPARED services?” Facilitators also completed a three-item subscale that assessed Perceived Effectiveness (i.e., whether the intervention met needs of adolescents) and a three-item Individual Professionalism subscale (i.e., self-efficacy in their role). Example item: “Is PREPARED a good way to address youths’ problems?” For all items on the subscales, respondents were asked to rate each item using a four-point Likert scale: “Not at all” (0), “A little bit” (1), “A moderate amount” (2), and “A lot” (3). All subscale scores were calculated by averaging responses across items.

Fidelity was measured through facilitator adherence to the intervention manual and was rated live by research assistants using detailed session checklists for each step. These checklists were based on steps outlined in the intervention manual, including didactic sections and facilitation of discussions and activities. They were rated based on if the step was completed fully, partially, or not at all. Facilitators were rated on only sections they were assigned to facilitate, so the number of steps varied by facilitator and session.

#### Implementation outcomes: qualitative FGDs

Qualitative FGDs were conducted post-intervention to further understand both adolescent and facilitator perceptions of implementation. Semi-structured interview guides were developed following the structure from previous pilot evaluations conducted in Kenya and the United States [48, 49] and included prompts related to intervention delivery, content, and engagement (provided in Supplemental Material C). Adolescent FGDs addressed the implementation as well as intervention-related changes (described below).

#### Preliminary effectiveness: survey measures in full sample

##### Positive well-being

Positive mental health attributes and well-being were assessed with the Warwick-Edinburgh Mental Well-being Scale (WEMWBS) [50]. Adolescents responded to 14 items on a 5-point scale from “none of the time” (1) to “all of the time” (5). Responses were summed with higher scores indicating greater well-being (α = 0.70).

##### Depressive symptoms

Depressive symptoms and severity were assessed using the Patient Health Questionnaire-9 (PHQ-9) [51]. Adolescents responded to nine items on a 4-point scale: "not at all” (0) to “nearly every day” (3). Responses were summed with higher scores indicating more depressive symptoms (α = 0.80). If an adolescent endorsed suicidal ideation (item 9), they were flagged for further risk assessment and follow up by lead investigator (SJ).

##### Psychological distress

Psychological distress was measured with the six-item Kessler Psychological Distress Scale (K-6) [52]. Participants responded on a 5-point scale: “none of the time” (1) to “all of the time” (5). Responses were summed with higher scores indicating greater psychological distress (α = 0.74).

##### Substance use

Adolescent substance use was assessed using an adapted version of the Brief Screener for Tobacco, Alcohol, and other Drugs (BSTAD) [53]. Respondents answered “yes” (1) or “no” (0) about past month use of tobacco, marijuana, alcohol, prescription pills, khat (a commonly used local drug), and inhalation of glue. Substance use was reported individually for each substance.

##### Social support

Perceived social support was assessed with the Multidimensional Scale of Perceived Social Supports (MSPSS) [54] Participants responded to 12 items using a five-point scale: “not at all” (1) to “a lot” (5). Responses were summed, with higher scores indicating greater perceived social support (α = 0.77).

##### Positive sexuality

Positive sexuality attitudes were measured using 14 items from the Positive Sexuality in Adolescence Scale (PSAS) [55]. Participants responded to the following subscales: positive approach to sexual relationships (seven items), resilience against challenging sexuality-related experiences (five items), and control over sexual interactions (two items). Participants responded on a 7-point scale, from “strongly disagree” (1) to “strongly agree” (7). Responses were averaged, with higher scores indicating greater agreement with positive approaches to sexual relationships (α = 0.85).

##### Acceptance of IPV

Acceptance of male-perpetrated physical IPV was assessed using the Violence subscale of the Gender Equitable Men scale (GEM) [56] adapted for the purpose of this study in adolescent relationships by including the language of boyfriend and girlfriend instead of husband and wife. The six items of the subscale measured an individual’s attitudes toward male-perpetrated violence. Participants responded on a 3-point scale from “agree” (1) to “disagree” (3). Responses were averaged, with higher scores indicating less acceptance (greater disagreement) of violence (α = 0.71).

#### Preliminary effectiveness: outcomes in subsample of adolescents in dating relationships

##### Relationship communication

Adolescent relationship communication was assessed using the 7-item Relationship subscale from the Couples’ Communication Scale (CCS) [57]. This measure was developed for both adult and adolescent relationships and includes items like “I express my feelings to my partner when I am upset with him or her.” Participants responded on a 6-point scale from “strongly disagree” (1) to “strongly agree” (6). Responses were averaged, with higher scores indicating better communication (α = 0.71).

##### Relationship power dynamics

Decision-making power dynamics relationships were assessed using the Decision-Making subscale of the Sexual Relationship Power Scale (SRPS) [58] that was adapted for adolescent relationships. The subscale is typically scored with “Your Partner” (1), “Both of You Equally” (2), and “You” (3). Given the interventions’ goals of shared decision making, responses were dichotomized such that Both of You Equally” was scored (1) and “Your Partner” and “You” were both scored (0) (α = 0.72).

##### Dehumanization in relationships

Dehumanization in romantic relationships (e.g., contempt, hostility, and conditional regard toward partner) was assessed using the Dehumanization in Romantic Relationships Scale (DIRSS) [59]. Of the 12 total items, only six were used for this study to reduce participant burnout. Three measured how one treats their partner (α = 0.80) and three evaluating how one is treated by their partner (α = 0.59). Participants responded to these items on a 7-point Likert scale ranging from “strongly disagree” (1) to “strongly agree” (7). Responses were averaged, with higher scores indicating greater dehumanization.

##### **Sexual risk behaviors**

Sexual risk behaviors were evaluated with a 6-item scale developed for this study. One item was removed due to lack of clarity. Participants responded “yes” (1) or “no” (0) to items like “I use a condom during sex” and “I have more than one sexual partner currently.” We report scores at the item level.

##### Victimization and perpetration of IPV

Victimization and perpetration of IPV was assessed using an adapted version of World Health Organization’s Violence Against Women Instrument (VAMI) [60, 61]. The 22-item measure has victimization and perpetration scales, each with three subscales for psychological (3 items), physical (6 items), and sexual (2 items) IPV. Participants responded “yes” (1) or “no” (0) to items. At baseline, lifetime and past month experiences were assessed. At endline, participants in relationships were asked about their experiences with their current partner “since starting PREPARED.” Binary subscale scores were calculated to reflect rates of each type of violence (i.e., experience of any of the three psychologically violent items = 1, experience of no psychologically violent items = 0).

#### Preliminary effectiveness: qualitative FGDs

During the adolescent FGDs, participants were asked about changes, if any, related to their individual well-being, beliefs about dating relationships, and approaches to sexual and dating relationships. They were probed about what led to specific changes, in hopes of elucidating potential mechanisms of change.

### Data analysis

#### Implementation outcomes

​​Descriptive and summary statistics were computed in R (version 2022.07.1; [62] to report facilitator and adolescent ratings on the implementation surveys and attendance rates. Fidelity results were calculated for each facilitator by determining the total percentage of accurate completed steps. Facilitator and adolescent qualitative FGDs were audio recorded and transcribed verbatim from Luo or Kiswahili to English.

Our qualitative analysis approach was guided by the steps of thematic content analysis [63]. First, transcripts were reviewed by members of the Duke research team to become familiar with the data. The facilitator codebook included deductive codes based on the Implementation Outcomes Framework [46] and the FGD guide related to general experiences of being a facilitator, perceptions of intervention content/materials, perceived effectiveness, and feedback on implementation and delivery. The adolescent implementation codebook included codes related to satisfaction, engagement, and skill use. Transcripts were analyzed with the support of NVivo (version 12) [64] based on initial code books. Each FGD was coded by at least two research team members, and code summaries were written for each code. Code summaries were then reviewed and collated into specific themes to clarify barriers and facilitators of successful implementation by the lead author (SJ).

#### Preliminary effectiveness

Descriptive and summary statistics were used to describe the demographics and history of adversity. With the pre-post survey data, we first evaluated the reliability of each measure. Measures with very low alpha estimates (< 0.50) or with substantial missingness that resulted from confusion or lack of understanding of items were not included in the primary analyses. For information about these excluded measures, please see Supplemental Material D. We then calculated the mean pre and post intervention scores for each of the variables to observe the direction of changes. Next, as preliminary indicators of intervention change, we estimated the effect size of changes with a calculation of Hedges’ *g* (paired)*—*a bias-corrected measure of Cohen’s *d* that indicates how many standard deviations the means differ [65]. Given the preliminary nature of this study and our limited sample size, we are prioritizing the estimation of effect sizes rather than hypothesis and significance testing and report confidence intervals. As an exploratory analysis, we also describe mean pre- and post- scores by implementation setting (school-based and community-based) and by sex (findings presented in Supplemental Materials E and F). All statistical analyses were completed in R (version 2022.07.1) [62].

**Fig. 2:**
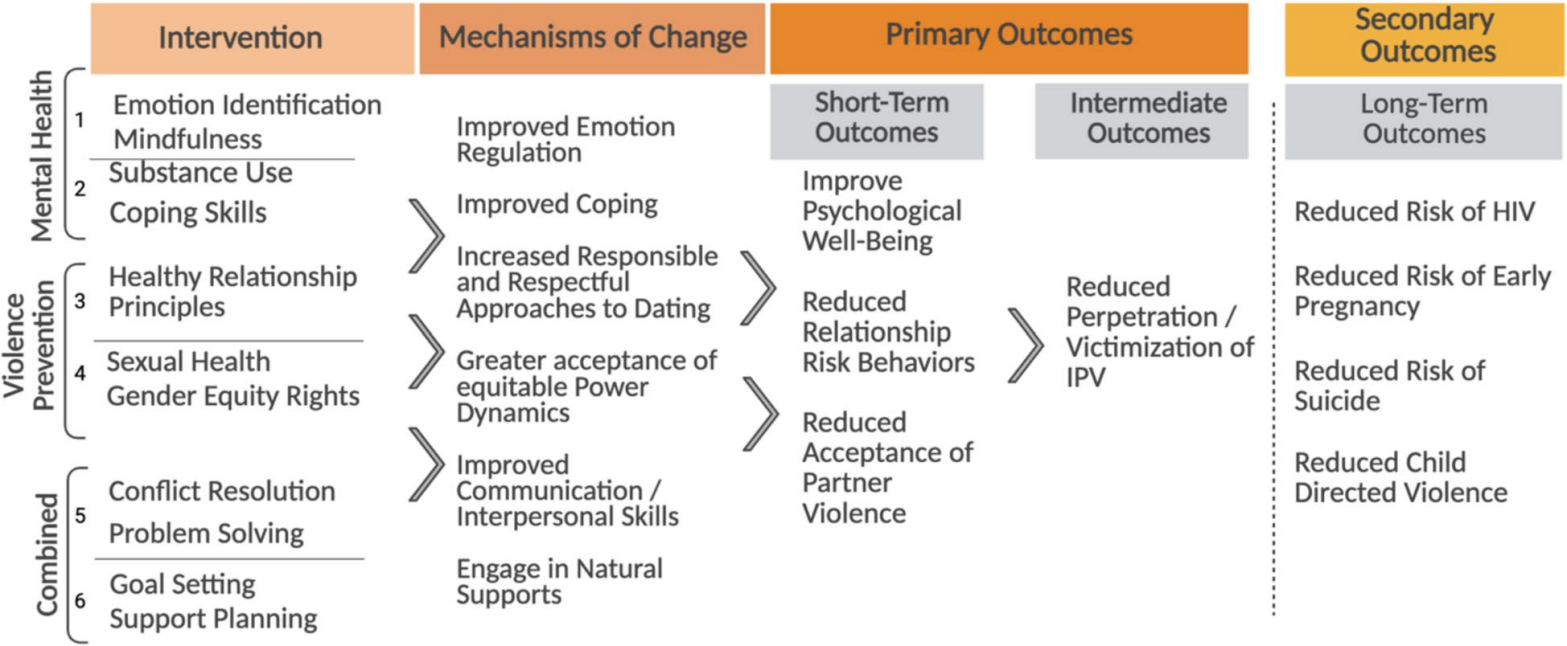
PREPARED hypothesized theory of change

Like our implementation FGD data, our qualitative analysis approach for intervention-related qualitative data was guided by thematic content analysis [63]. The development of the effectiveness outcomes codebook was guided by our hypothesized theory of change (Fig. 2), and the analysis mirrored the implementation FGDs described above.

## Results

### Participants

Demographics of participants and facilitators are reported in Table 2. Intervention participants (*N* = 46) had a mean age of 15.43 years (*SD*** = **1.09), with 24 (52%) identifying as female. Most participants’ biological parents acted as their caregivers (*n* = 32, 67%) and, on average, their households consisted of seven family members. The vast majority identified as being Christian (*n* = 44, 96%). Half were in a dating relationship at baseline (*n* = 22) and nearly half reported being sexually active (*n* = 21). Five females reported having a child of their own. The mean ACE score was 6.65 (out of 10). On average, across both the community and school groups, youth attended 5.5 sessions out of 6 sessions and 33 youth (72%) attended all 6 sessions. Demographics are also reported by the implementation setting in Table 2. Notably, the out-of-school (community-based) group included more participants who were in relationships, were sexually active, had children of their own, and reported greater exposure to ACEs. For the FGDs, all 22 school-based adolescents participated and 20 (83%) of the community-based adolescents participated.

**Table 2:** Demographic characteristics of participants and facilitators

	Total ***N*** = 46		School-Based Group ***N*** = 22		Community Group ***N*** = 24	
	***M or N(%)***	***SD***	***M or N(%)***	***SD***	***M or N(%)***	***SD***
Participants						
Age	15.43	1.09	15.18	0.91	15.7	1.20
Sex (Female)	24 (52)	0.51	12(55)	0.51	12 (50)	0.51
Caregiver (Biological Parent)	31(67)	0.47	14 (64)	0.49	17(71)	0.46
Number of Household Members	7.21	3.27	7.14	3.00	7.3	3.57
Religion (Christian)	44 (96)	0.21	22 (100)	0.00	22 (92)	0.28
Relationship Status (In Relationship)	23 (50)	0.50	4 (19)	0.40	19(79)	0.41
Sexually Active (Yes)	21 (46)	0.50	3 (14)	0.35	18(75)	0.44
Children (Has a child)	5 (11)	0.31	0 (0)	0.00	5 (20)	0.41
Adverse Childhood Experiences	6.64	2.53	5.68	2.44	7.54	2.32
Sessions Attend	5.5	1.05	5.68	0.78	5.33	1.24

	Total *N* = 10		School-Based Group *N* = 5		Community *N* = 5	
	*M* or *N*(%)	*SD*	*M or N(%)*	*SD*	*M or N(%)*	*SD*
Facilitators						
Age	21.2	1.93	21.8	2.2	21	1.4
Sex (Female)	10 (50)	0.53	2 (40)	0.55	3 (60)	0.55
Education Level (Completed Secondary)	10 (100)	0.0	5 (100)	0.0	5 (100)	0.0
Training Days Completed	11.6	0.52	11.6	0.55	11.6	0.55
Sessions Facilitated	5.7	0.67	6	0	5.4	0.55

Facilitators (*n* = 10) had a mean age of 21.2 years (*SD* = 1.93). There were 5 male and 5 female facilitators, all of whom had completed secondary school. None were married or had children. Six facilitators attended all 12 training days, and the remaining four attended 11 of the 12. Seven facilitators attended all six intervention sessions, and the remaining three attended five of the six. Overall fidelity to the intervention was high ranging from 83 to 100% across facilitators and sessions; most ratings were above 95% and the mean was 97.8%.

### Implementation outcomes

#### Quantitative survey data

Adolescent and facilitator reports of implementation indicated support for the intervention’s adoption, acceptability, appropriateness, feasibility, and reach/access. Facilitator responses suggest alignment with their professional identities and support of the intervention’s perceived effectiveness. Results are presented in Table 3, including results by implementation setting. Results were generally consistent across the two implementation settings; however, facilitators reported lower feasibility in the community-based implementation (*M* = 2.56*, SD* = 0.70) compared to school-based (*M* = 2.96*, SD* = 0.06). Across all outcomes, mean ratings from full samples of adolescents and facilitators were 2.66 and above out of three.

**Table 3:** Implementation outcomes

Participants	Range	Full Sample ***N*** = 46		School-Based ***N*** = 22		Community-Based ***N*** = ***24***	
		Mean	SD	Mean	SD	Mean	SD
Adoption	0–3	2.78	0.31	2.86	0.31	2.71	0.30
Acceptability	0–3	2.88	0.22	2.84	0.27	2.91	0.16
Appropriateness	0–3	2.89	0.21	2.89	0.26	2.90	0.16
Feasibility	0–3	2.88	0.21	2.81	0.26	2.94	0.12

Facilitators		Full Sample *N* = 10		School-Based *N* = 5		Community-Based *N* = 5	
	Range	Mean	SD	Mean	SD	Mean	SD
Adoption	0–3	2.81	0.22	2.71	0.27	2.91	0.13
Acceptability	0–3	2.94	0.09	2.93	0.11	2.95	0.07
Appropriateness	0–3	2.66	0.53	2.76	0.36	2.91	0.13
Feasibility	0–3	2.98	0.05	2.96	0.06	2.56	0.70
Reach & Access	0–3	2.92	0.25	2.96	0.06	2.84	0.36
Professional Identity	0–3	2.97	0.11	3.00	0.00	2.93	0.15
Effectiveness	0–3	3.00	0.00	3.00	0.00	3.00	0.00

#### Qualitative results

Qualitative outcomes further clarified participant and facilitator perceptions of implementation. We first present implementation themes shared across participant and facilitator discussions, then present themes that are distinctly defined by participant or facilitator perspectives.

#### The content of the intervention was generally relevant and acceptable to participants and facilitators, with adaptations needed related to conflict resolution and condom negotiation

Overall, the specific content and materials of the intervention were well-received by adolescents and easily employed by the facilitators. The session skits and demonstrations were the most engaging parts for the adolescents and were also the facilitators’ favorite components to lead. The metaphors used in the content were helpful for explaining new concepts, especially pertaining to mental health. For example, one youth shared,

*“I loved the bit of the bottle metaphor. The demonstration made so much sense in that one could easily understand that when we pile up problems [on] ourselves, we will eventually explode and when we release the pressure inside us slowly, we come out of the problems without exploding.” -Male Adolescent, Community-Based Group*

The conflict resolution skills were identified as the most confusing by both adolescents and facilitators. The process presented included multiple steps and was introduced later in the intervention. Following multiple steps, and limited time for skills practice, may have led to less skill use among participants. There was also mixed feedback from both participants and facilitators regarding the condom use negotiation skills content. Most adolescent females found it useful, especially “learn[ing] how to use both female and male condoms.” In contrast, adolescent males found this content unhelpful and reported that peers outside of the program disagreed with what they learned about condom use (e.g., sex could be pleasurable with a condom). Facilitators also mentioned some discussions about condom use were difficult with youth who did not intend to change behaviors.

#### The structure of the intervention was appropriate, but youth expressed interest in the program continuing and facilitators suggested a need for more sessions

Adolescents seemed to appreciate that some portions of the sessions were delivered with males and females combined in a larger group and some portions were delivered to sex-specific groups. They seemed to especially appreciate that more sensitive topics were first discussed in the smaller sex-specific groups. Regarding length of the program, most adolescents reflected that the intervention should be longer and extended, both in the number of sessions and length of individual sessions. Some reported that they wished there were more sessions so the groups could continue to gather and learn together. It was also suggested that the program should continue in general because “these lessons should reach as many youths as possible.”

Facilitators suggested that more time was needed to cover some of the topics. Sessions 4 and 5 (related to healthy relationships, problem solving, conflict resolution) were identified as a session that could be broken up into more sessions given, they extended beyond the planned two hours. This may have been why these skills were identified as the most difficult given time limitation seemed to be a factor. This is consistent with conflict resolution skills being those that were reported to be most difficult to apply. One facilitator shared,

*“If the session has a lot of information at the same time it’s hard to deliver the whole of it within the two hours. So maybe [if] we have one more session, there would be enough time for this program.” -Male Facilitator*

#### Adolescents connected with the young adult facilitators, and facilitators were motivated by their support of the adolescents

Adolescents reported great satisfaction with facilitators. Many youth reported finding them “helpful” and “understanding” of their challenges. Facilitators being perceived as relatable helped the adolescents open in sessions and motivated their continued engagement with the program. One youth reflected,

*“We had good facilitators who were committed to helping us. They gave us advice on issues that we felt were too heavy for some of us and helped us learn.” -Male Adolescent , Community-Based Group*

Facilitators were motivated by the opportunity to build relationships with youth and teach them skills that could “make a difference.” The content related to developing healthy dating relationships seemed to be especially meaningful to share with the youth. Also, their proximity in age to the participants cultivated greater connection.

*“I also hoped that the youth could learn from what I was going to teach because they are just like me, a youth. A problem shared is halfway solved.” -Female Facilitator*

#### Community-based youth appreciated being reached in the community while the school-based adolescents enjoyed participating in the program at school

The youth in the community-based setting were not enrolled in school which limited their opportunities to interact with other adolescents and to engage in learning new content. One youth reflected that he had “not been in class for a very long time” and “PREPARED has enabled [me] to try to remember writing and reading.” Both male and female participants in this group were grateful to be engaged and reached outside of the school setting.

*“It was a good idea to [also] have the program in the community because many youths have benefited from it by being able to learn.” -Female Adolescent, Community-Based Group*

The youth in the school-based setting reflected that participating in the intervention at school was “safe” and “comfortable.”

#### Facilitators reflected their own professional and personal growth after being selected and trained as a facilitator

Being involved in the intervention as a facilitator seemed to enhance both professional and personal development. Many facilitators shared a sense of self-efficacy when reflecting on their newly learned skills. They reported confidence in applying the skills in the sessions as well as with neighbors and family members. One even reflected he felt he is now in “a better position in the community.” Another reflected,

*“PREPARED has improved my way of interacting with people...I learned that I had to create a good rapport with the youths and be friendly with them. I learned that I had to be talkative and be able to articulate myself well while facilitating. I am now a strong person who can address a crowd without any fear.” -Male Facilitator*

### Preliminary effectiveness

We report descriptive statistics for pre-and post-survey data and effect sizes to describe the magnitude of change of the primary outcomes in the full sample (*N* = 46) in Table 4. Relationship-related outcomes for adolescents who were in the same romantic relationships over the course of the intervention (*n* = 20; 43% of overall sample) are also reported in Table 4. Results provide preliminary evidence for some positive changes at both the individual and relationships levels.

**Table 4:** Preliminary effectiveness outcomes

Outcomes	Possible Range	Valence	Pre-Mean (*SD*)	Post-Mean (*SD*)	Hedges’ *g*	95% CI
Primary Outcomes (*N* = 46)						
Well-Being	7–35	+	23.37 (5.0)	26.57 (5.34)	0.61	0.18, 1.04
Depressive Symptoms	0–27	-	8.32 (5.03)	6.63 (5.26)	−0.32	−0.75, 0.11
Psychological Distress	0–24	-	9.46 (5.03)	8.11 (3.91)	−0.34	−0.77, 0.10
Perceived Social Support	7–84	+	59.14 (12.49)	66.87 (11.31)	0.64	0.23, 1.04
Violence Attitudes (Disagreement)	1–3	+	2.28 (0.56)	2.39 (0.58)	0.19	−0.28, 0.67
Positive Sexual Relationships	1–7	+	5.05 (1.21)	5.93 (0.97)	0.78	0.24, 1.32
Relationship Outcomes (*N* = 20)						
Couples' Communication	1–6	+	5.01 (1.02)	5.48 (0.65)	0.51	−0.05, 1.08
Shared Decision Making	0–1	+	0.36 (0.28)	0.49 (0.21)	0.51	0.02, 0.99
Dehumanization by Partner	1–7	-	2.85 (1.85)	2.05 (1.66)	−0.40	−1.05, 0.24
Dehumanization of Partner	1–7	-	2.67 (1.44)	2.07 (1.41)	−0.44	−1.01, 0.14

#### Preliminary effectiveness outcomes in full sample

In the full sample, pre-post changes are in the expected direction across mental health outcomes. The largest mental health effect, in the medium sized range, is observed on adolescent well-being. The mean score increased from 23.37 at baseline to 26.57 after the intervention (Hedges’ *g* = 0.61; CI: 0.18, 1.04).

Depressive symptoms and psychological distress both decreased, with small effect sizes (Hedges’ *g* = −0.32 and −0.34 respectively, both non-significant). Substance use results are not presented in the table given very low reported use at baseline and endline; only 5 youth reported any substance use at pre- and/or post-test. Average perceived social support increased from 59.14 (out of 84) to 66.87 post intervention, a medium effect size (Hedges’* g* = 0.64; CI: 0.23, 1.04).

Regarding attitudinal shifts, there was already moderate disagreement with male-perpetrated violence at baseline, with essentially no change post intervention. There was, however, a medium sized effect observed on positive approaches to sexual relationships (i.e., control, mutual respect, and resilience against challenges in sexual relationships) after the intervention with average agreement increasing from 5.05 (out of 7) to 5.93 (Hedges’* g* = 0.78; CI: 0.24, 1.32).

Exploratory analyses of pre-post changes by implementation setting and sex are presented in Supplemental Materials E and F. The observed mental health effects were stronger in the school-based group compared to the community-based group, especially for changes in well-being and depressive symptoms. However, the community-based group reported more depressive symptoms and slightly greater psychological distress compared to the school-based group. The observed effect of perceived social support was also greater in the school-based group. There was a stronger effect on agreement with positive approaches to sexual relationships in the community-based group.

Results by sex also revealed some important patterns. Larger changes were observed among female participants across mental health outcomes (well-being, depressive symptoms, and psychological distress). Female participants also developed somewhat greater disagreement with male-perpetrated violence compared to male adolescents, whose attitudes did not change at all. For male participants, however, there was a greater observed effect on agreement with positive approaches to sexual relationships compared to female participants.

#### Preliminary effectiveness outcomes in sample of sexually active adolescents

At baseline, 21 of 46 adolescents (~ 46%) reported being sexually active. From pre-test to post-test, all sexual behavior outcomes changed in the expected direction, though changes were small. Specifically, participants reported reducing their sexual partners to only one (decreasing from eight to three participants), slightly higher condom use (increasing from 11 to 13 participants), elimination of receiving payment for sex (decreasing from two to zero), reduced payment for sex (decreasing from two to one), and increased HIV/STI testing (increasing from 13 to 16 participants). A table of these findings are in the Supplemental Material G.

#### Preliminary effectiveness outcomes in sample of adolescents in dating relationships

Results from adolescents who were in the same dating relationship over the course of the intervention are presented in Table 4. The mean age of this sample was 15.9 (*SD* = 1.12) and the reported mean age of partners was 17.6 (*SD* = 2.46); three adolescents (15%) reported having a partner 5 or more years older than them. Among participants who reported that they were in the same dating relationship at both pre and post (*N* = 20), openness in couples’ communication was already high at baseline (*M* = 5.01 out of 6) but still improved further after the intervention with an average score of 5.48 indicating a moderate effect (Hedges’ *g* = 0.51, non-significant). A moderate effect was also observed on equitable decision-making power (Hedges’ *g* = 0.51; CI: 0.02, 0.99). There were small effects on dehumanization in romantic relationships; participants' belief that their partner dehumanizes them and a participant’s dehumanization of their partner both decreased (Hedges’ *g* = −0.40, −0.44 respectively, both non-significant).

Overall, lifetime experience of IPV victimization and perpetration by a current or previous partner was common. Specifically, 19 of the 20 adolescents (95%) reported psychological victimization, 17 (85%) reported physical victimization, and 17 (85%) reported sexual victimization. All 20 (100%) reported at least one type of victimization. Regarding perpetration, 17 adolescents (85%) reported psychological perpetration, 18 (90%) reported physical perpetration, and 14 (70%) reported sexual perpetration. Almost all (19; 95%) reported some type of perpetration.

Pre-post changes to IPV are presented in Table 5. To explore changes, we present *past month* reports of IPV as baseline and reports of IPV *since the PREPARED intervention began* at endline. Two fewer participants reported physical victimization at endline (17 to 15) and one fewer reported sexual victimization (14 to 13) at endline. In terms of IPV perpetration, two fewer participants reported psychological perpetration (16 to 14) and physical perpetration (17 to 15) and one fewer reported sexual perpetration (14 to 13) at endline. Both male and female participants reported victimization and perpetration pre and post intervention as presented in Table 5.

**Table 5:** Pre-post intimate partner violence outcomes among adolescents in dating realtionships

IPV Outcome	Participants in Dating Relationship N = 20		Female N = 10		Male N = 10	
	Pre N (%)	Post N (%)	Pre N	Post N	Pre N	Post N
Victimization Psychological	16 (80)	16 (80)	8	9	8	7
Victimization Physical	17 (85)	15 (75)	9	8	8	7
Victimization Sexual	14 (70)	13 (65)	7	7	7	6
Perpetration Psychological	16 (80)	14 (70)	7	7	9	7
Perpetration Physical	17 (85)	15 (75)	9	7	8	8
Perpetration Sexual	14 (70)	13 (65)	6	7	8	6

#### Qualitative results

We present qualitative themes that reflect positive changes in individual coping, engagement in social supports, and relationship processes. 

#### Participants used new skills to engage in emotion identification and regulation, especially feelings of anger

Both adolescent males and females reflected on changes in their ability to identify and notice their feelings. This emotion identification seemed to set the stage for adolescents to engage in emotion regulation, especially for anger. A relaxation skill introduced during session 1, “Mountain and Valley Breathing” is practiced in each of the six sessions and was commonly identified as the skill used outside of sessions to address feelings of anger, stress, or frustration. For example, one participant expressed,

“Before attending this program, I used to get angry with people or even myself *to the extent that I don’t talk to anyone, but this program has taught me that I can solve my own issues using certain skills like mountain and valley breathing.” -Female Adolescent, Community-Based Group*

#### Changes within adolescent relationships varied but highlighted improvements in communication

Participants in relationships reflected some healthier dynamics in dating relationships, especially regarding communication. There was evidence of more equitable conversations, with one adolescent female sharing she now has the confidence to “look her boyfriend in the eye” when she speaks to him. Both male and female adolescents reference using their communication skills (Conversation, Listening, Encouragement, Appreciation, and Respect “CLEAR”) while community and while resolving differences.

*"My most interesting part was the CLEAR Skills. I learned that my partner and I should be able to Converse first before doing anything, Listen to each other, Encourage each other, Appreciate each other and Respect each other.” -Female Adolescent, Community-Based Group*

There were some positive changes related to sexual risk behaviors in relationships including no longer having more than one partner after learning that is “not safe for all of us.” Another youth shared that the intervention prompted him to be tested for HIV:

*“I learned that it is important for my girlfriend and I to go test before we engage in any sexual activity. I did my first test after this session, and it was really helpful.” -Male Adolescent, Community-Based Group*

Changes in condom use and attitudes toward condoms were mixed. While some adolescent females seemed to feel empowered with new knowledge and skills, other adolescent females and most adolescent males reflected that condom use was the one PREPARED skill they are not using in their lives outside of the intervention.

#### Participants expressed greater openness to seek support

Adolescents in both the school-based and community-based groups, reflected a greater trust in the people around them and an increased openness to seeking support. Sources of support include peers, caregivers, elders, and for the school-based group, their teachers. Adolescents reported applying the “Talk It Out” (i.e., identifying a trusted person and making a plan to talk to them) skill within these different relationships. At the peer level, an adolescent female in the school-based group shared she can now “Talk it Out with trusted friends.” Another youth shared,

*“Amongst peers, we are able to assist each other in case of a problem that one of us faces. We combine efforts to give advice until our fellow peer feels better.” –Male Adolescent, community-based group*

Adolescents also shared being able to “open up” to their parents for the first time, and in some cases, listen to their parents’ or caregivers’ advice and support. It also seemed seeking support from caregivers improved the relationship quality between adolescents and their caregivers. One adolescent shared,

*“I used not to listen to my mum or understand her when she told me that there isn’t enough money to pay for my education. I used to cry, distance myself, and not talk to her. With the help of PREPARED, my relationship with my mum has improved. We help each other and have become best of friends.” -Female Adolescent, Community-Based Group*

For the school-based group, they expressed greater engagement in support from their teachers. Changes included seeking emotional support from teachers as well as gaining the confidence to express other needs. This is promising given the amount of time many of the youth in this group spend at school with their teachers.

​​ “Before I couldn't go to ask the teacher concerned for sanitary pads but now I can *because of the confidence I gained from the program.” -Female Adolescent, School-Based Group*

## Discussion

Our proof-of-concept study of the PREvention Program for Adolescent Relationship and Emotional Development (PREPARED) evaluates implementation outcomes and preliminary intervention changes in a low-resource setting. PREPARED is a novel approach to mental health promotion and violence prevention, aimed at improving individual coping and healthy romantic relationship development. Overall, our preliminary assessment of implementation outcomes supports successful task-shared delivery in both a school and a community setting. Measures of intervention change are largely promising for mental health outcomes, while relationship-related outcomes are mixed.

Based on the Implementation Outcomes Framework [46], the implementation of PREPARED proved successful in both school and community contexts. The involvement of the CAB, from the formative stages through pilot implementation and interpretation of findings, provided the foundation for the culturally responsive content and approach. The intervention itself, designed by pairing evidence-based principles of mental health promotion and violence prevention with culturally tailored content, was generally appropriate and well-received in this setting and by the target population. The implementation plan and protocol were defined by the community members which allowed for careful consideration of potential barriers to reaching adolescents and ensuring the program was accessible. Together, this study contributes to the real promise of the principles and goals of CBPR in developing and evaluating an intervention—especially one that addresses such sensitive topics [42, 66].

Our near-peer, non-specialist delivery was completed with high fidelity, building upon successful approaches for task-shared delivery in LMICs [67] and other peer provider models for adolescent interventions in Kenya [68, 69]. The selection of these facilitators, for their proximity in age and social characteristics to adolescent participants, promoted connection, trust, and relatability. Their “social proximity” was beneficial for both adolescents and facilitators in our study, consistent with other peer-delivery models [24, 70, 71]. While the facilitator to adolescent ratio was ~ 1:5 in this pilot, findings suggest we could test ratios of 1:10 to 1:15 in larger implementations for greater feasibility—especially as we had two lead facilitators in each setting and the other facilitators played more supportive roles.

Regarding the implementation settings, facilitators did find the school-based implementation slightly more feasible based on survey findings, and the youth found it beneficial to embed the implementation in a familiar setting where adolescents already gather for their education. However, the community-based implementation extended the study’s reach by engaging youth often missing from intervention efforts. Given 258 million adolescents are not enrolled in school and miss out on school-based and health promotion efforts, it is critical to develop strategies for reaching these often overlooked youth [72]. Our baseline data show greater mental health needs and less perceived social support among out-of-school youth, who were also more likely to be in dating relationships, sexually active, and parents.

Our pre-post survey results, while promising, must be cautiously interpreted due to the small sample and lack of control group. Findings indicated improved well-being, fewer depressive symptoms, and lower psychological distress, with small to medium effect sizes. Based on qualitative findings, PREPARED engaged emotion identification and emotion regulation processes, effective elements for mental health promotion and resilience building in other interventions in LMICs [73, 74]. Adolescents also reported greater perceived social support, qualitatively reflecting more engagement with peers, parents, and teachers for emotional support, which can be protective against depression and anxiety in adolescents in Kenya [75]. The enhanced communication with parents and guardians is particularly promising given the protective role of parental involvement against psychological distress and mental health problems in LMICs [76].

Regarding relationship attitudes, our results were mixed. Adolescents in relationships showed greater agreement with shared satisfaction and control in relationships, but in the full sample, there was no change in attitudes toward male-perpetrated physical violence. At baseline, there was already moderate disagreement with male-perpetrated violence, perhaps leaving little room for shifts. Exploratory analysis by sex revealed a small shift among females towards greater disagreement with violence, whereas males showed no change. Taken together, the intervention appeared to engage more nuanced beliefs about specific aspects of relationships but did not alter attitudes towards relationship violence. Attitudes toward violence may be more deeply influenced by community-defined norms, requiring larger scale and longer-term shifts in the community like the *SASA!* community mobilization intervention in Uganda which led to lower acceptance of IPV among men and women [77]. Future efforts may benefit from more gender transformative content and prompts for critical reflection and engagement [17]. Interestingly, there is counterintuitive evidence from SSA suggesting acceptance of IPV has protective mechanisms for girls and women who have experienced IPV [78]. This might be due to cognitive dissonance where there is a discrepancy between a person’s beliefs (e.g., disagreement with violence) and reality (e.g., victim of violence; [78, 79]).

While improvements were noted in communication and equitable decision-making among youth in relationships, there were no substantial changes in IPV victimization or perpetration. The intervention may not be intensive enough to reduce immediate ongoing violence; however, observed improvements in communication and decision making, along with improvements to individual mental health, are potentially precursory changes that could lead to longer-term reductions in IPV [80].

### Strengths and limitations

Strengths of our study include being the first, to our knowledge, to evaluate an intervention combining mental health promotion and IPV prevention for adolescents. We collected implementation data from both facilitator and adolescent perspectives, providing insight at both levels. Additionally, we assessed victimization and perpetrations among males and females, rather than solely on female victimization in the literature from LMICs. Finally, in our pre-post survey and qualitative data collection we measured potential mechanisms of change—including well-being, perceived social support, openness to responsible approaches to dating relationships, communication skills—to help fill the mechanistic gaps in this literature [7]. Our proof-of-concept phase of this work, a step that is sometimes overlooked in intervention research, critically identifies benefits and potential harms before larger trials [43, 81].

There are also notable limitations. Our small sample size and pre-post design are not intended to test intervention effectiveness; thus, results are preliminary, hypothesis generating, and lay the foundation for future larger effectiveness trials. As with many pre-posttest designs, this study is subject to several threats to internal validity [82]. Regression to the mean is a particular concern given our short timeframe between assessments; participants with extreme scores at baseline may naturally show improvement at posttest regardless of effects from PREPARED. The lack of a control group prevents us from determining whether observed changes exceed what would be expected through natural variation or other concurrent factors. These limitations underscore the preliminary nature of our findings and the critical need for future research employing randomized controlled designs with adequate follow-up periods.

Further, there was a methodological limitation in how we measured IPV outcomes. At endline, we assessed IPV since the start of the intervention with the intention of trying to capture any exposure of IPV during the intervention. However, this created challenges in interpretation and comparability with the baseline measure. For future trials, we aim to have consistent timeframes at both baseline and endline as well as follow-up assessments at one-, three-, and six-month post-intervention to better measure changes on IPV outcomes.

There were also measurement concerns, with measures of coping and emotion regulation not performing well (low reliability) in addition to clarity or social desirability concerns related to self-report of relationship outcomes. Evidence for these concerns is valuable and points to the need for culturally emergent measures related to coping and distress [83], especially given existing evidence around the protective nature of religious coping with poverty and mental health in this setting [84]. Our study also did not test mental health and relationship changes over time, which is a goal of future longitudinal effectiveness trials.

### Implications for future work

Implementation and intervention results support PREPARED’s overall proof of concept, setting the stage for larger randomized control trials. Successful delivery by non-specialist providers shows potential for implementing similar interventions in rural settings with limited health infrastructure, especially those outside the United States and SSA where most IPV prevention evidence is drawn [7]. Given the nature of the content of this intervention and the use of near-peer providers not much older in age, it would also be helpful to measure changes to attitudes and behaviors around relationships at the facilitator level. This might also be able to speak to the potential for widespread community-level shifts around violence.

Given baseline rates of mental health challenges and rates of IPV, true promotion and prevention efforts need to occur *earlier* in adolescence (e.g., ages 10–13). Findings indicate this study served as a targeted prevention and early intervention model, rather than solely universal prevention. This is important given the greater fluidity of relationships during adolescence. Feedback suggested that some of the sessions, especially around relationship communication and sexual health, were too long for the two-hour period. Distilling essential content and considering opportunities for shortening the length of individual sessions by adding additional sessions—that specifically provide opportunities for participatory engagement around the violence prevention and sexual health elements—may be necessary based on evidence from other violence prevention interventions [15]. Finally, adaptations are needed to engage both adolescent females and males in content related to condom use and condom negotiation. Males in particular found the content around condom use and sexual health unhelpful and less engaging, yet there is great need to engage males in HIV prevention and reduction efforts as they are often missing [85, 86]. Such adaptations might include greater exploration of the myths related to condom use and work to explore behavior change in the context of one’s values and goals for their future. Based on previous literature, it might also be helpful to include opportunities for HIV testing—for girls and boys—within the intervention to help support prevention efforts [86]. Further adaptations are needed for the conflict resolution skill building, which might include adding additional practical application of the skills and opportunities for adolescents to participate in the co-creation of those skills through critical reflection and discussions based on their lived experiences [15].

## Conclusions

A combined mental health promotion and violence prevention intervention for adolescents was successfully delivered by non-specialist providers, demonstrating feasibility and acceptability. Preliminary findings suggest positive changes in mental health, social support, and approaches to relationships. There was no observed effect on IPV outcomes. Larger, fully powered randomized-control studies are warranted to assess long-term preventative effects on mental health and IPV among adolescents.

## Supplementary Information

Supplementary Material 1.

## Data Availability

The datasets used and/or analyzed during the current study are available from the corresponding author upon request.

## Abbreviations

IPV: Intimate Partner Violence
LMICs: Low- and Middle-Income Countries
HICs: High-Income Countries
Sub-Saharan Africa: SSA
FGDs: Focus Group Discussions
CAB: Community Advisory Board
CBPR: Community Based Participatory Research
ACEs: Adverse Childhood Experiences
