# Supplementary material for: The PREvention Program for Adolescent Relationship and Emotional Development (PREPARED): a proof-of-concept study
Source: BMC Public Health. 2026 Mar 16;26:1320. doi: 10.1186/s12889-026-26880-w (PMC13104496; doi:10.1186/s12889-026-26880-w)
Supplement: Supplementary file 1 — Supplementary Material 1. [file 12889_2026_26880_MOESM1_ESM.pdf]

Supplemental Materials

Supplemental Material A

Community Advisory Board Composition

Name	Age	Gender	Location	Occupation	Religious Affiliation
Grace Akoth	34	F	Southeast	Assistant Chief	Christian
Maureen Muchogo	38	F	East	Assistant Chief	Christian
Saleh Benard	40	M	West	Village Elder	Muslim
Miriam Anyango	44	F	Central	Assistant Chief	Seventh Day Adventist
Peter Ochieng	36	M	Central	Boat builder; Fishing Committee	Catholic
Hassan Job	33	M	East	Teacher (Primary); Pastor	Seventh Day Adventist
Faith Tambo Helleh	35	F	Central	Teacher (Secondary, Guidance & Counseling)	Christian
Kennedy Mikula	37	F	East	Teacher (Secondary)	Christian
Johnson K. Obiero	48	M	Central	Deputy Headteacher (Primary)	Christian
Elphalet Owino Ogwang	65	M	Southeast	Retired Chief	Seventh Day Adventist
Tenny Nyandwe Aluosh	15	F	West	Student	Christian
Fred Werema Kaiga	16	M	East	Student	Christian Union

Supplemental Material B

Participatory Mind Mapping

Intervention Development Work with Community Advisory Board

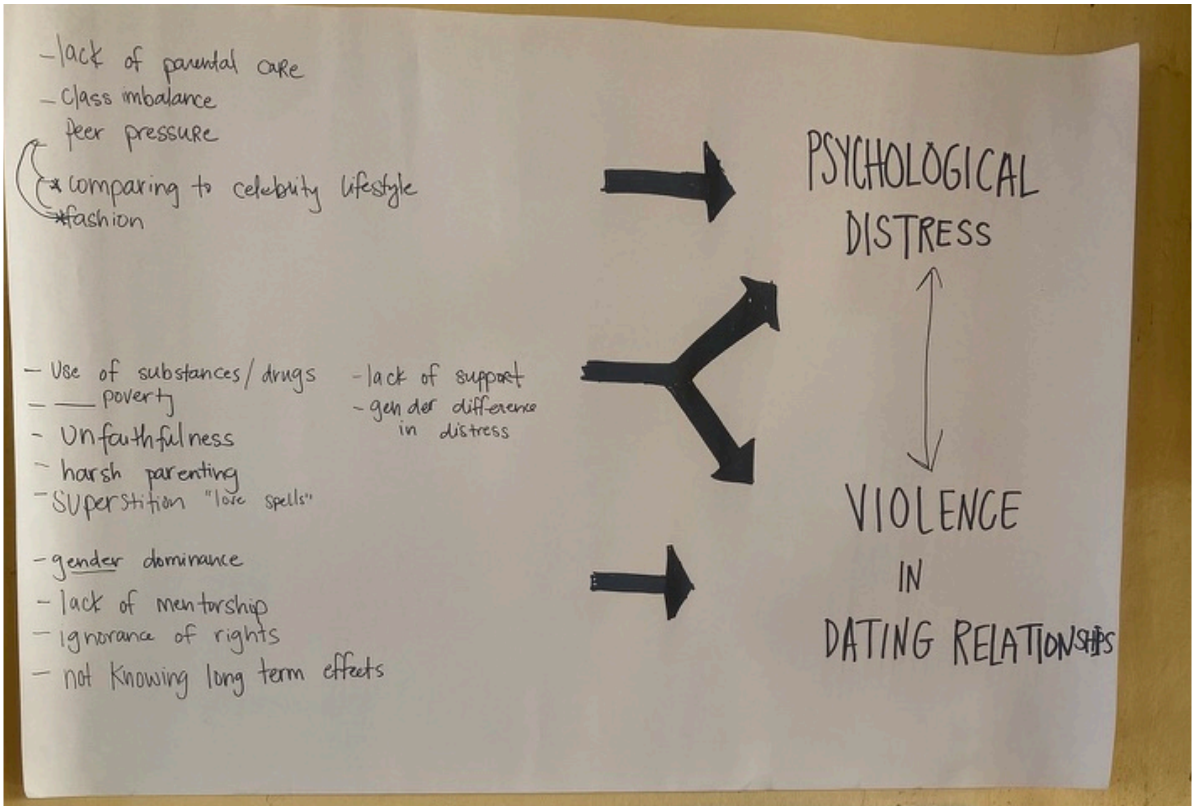

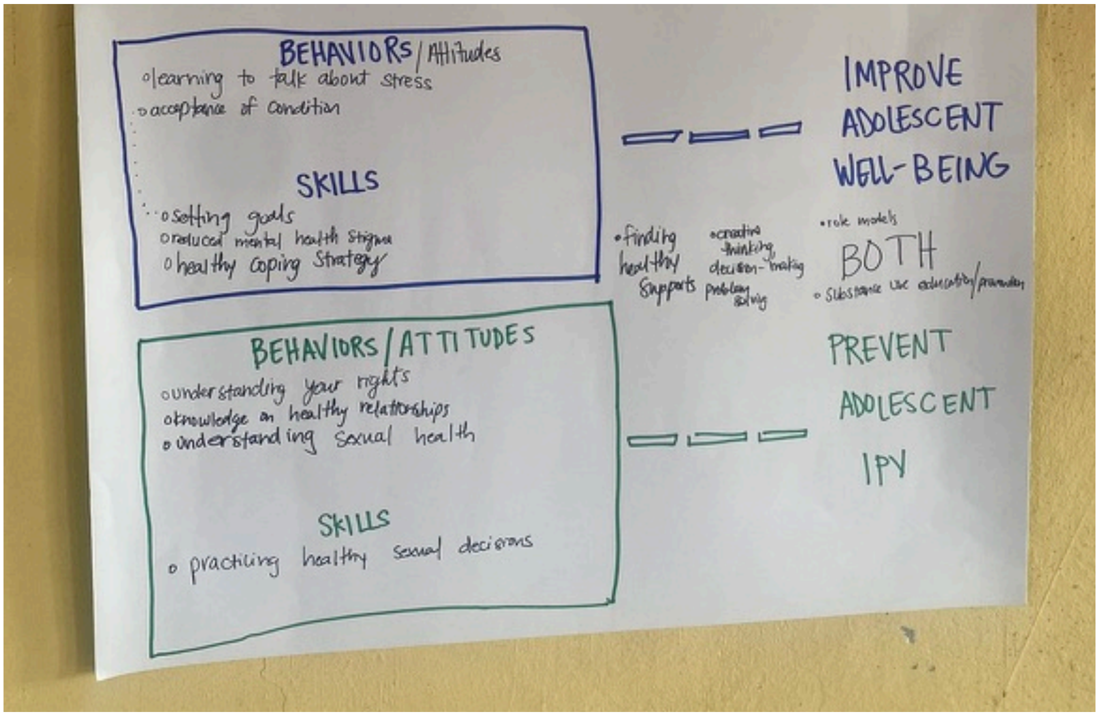

Supplemental Material C

Facilitator Endline Focus Group Discussion
Interview Guide

[English]

1) **Introduction and welcome (5 min)**
   a) Welcome and thank you so much for joining me today.
   b) I know you have already completed a survey and have given a lot of valuable feedback that will directly improve the PREPARED program. Thank you for that! Today, I wanted to take the opportunity to chat with you now that you're all finished with your groups, and maybe you have had some time to reflect on your experience.
   c) The purpose of our meeting today is to learn more about *your* unique experience as a facilitator and contributor to PREPARED. *You are the experts in how facilitators experience PREPARED.*
   d) I'll ask you some questions and I'd love to hear your honest thoughts and feelings on the program. Please be open and honest with me; you're not going to hurt my feelings and I fully expect that there are ways to improve. My goal is to make PREPARED better for the next group of families, and facilitators like yourself!

2) **Reflections on being a PREPARED Facilitator:**
   a) To start, I'd love to hear your overall reflections on what it has been like being a PREPARED facilitator.
   b) Why did you want to become a facilitator? What did you hope to get out of the experience?
   c) What were the most positive aspects, or your favorite parts, of being a facilitator?
      i) *After getting details about external factors related to their role, probe for some self-reflection:* What were the strengths YOU brought as a facilitator that made it a positive experience?
   d) What are aspects of being a facilitator that you did not like as much?
      i) *Probe: After getting details about external factors related to their role, probe for some self-reflection:* Were there any tasks or parts of facilitating that you struggled with?
   e) Are there any ways that PREPARED has changed aspects of your own life?
      i) Have you gained anything from PREPARED that you find yourself incorporating in your work or community roles?
      ii) What about in your own family?
      iii) Are there any changes in your own personal perspective on things?

3) **Reflections on PREPARED Content:**
   a) Now I'd like to ask about the lessons and skills that PREPARED includes. Each of the PREPARED sessions were meant to build on each other, so that youth could learn and grow through each step.

b) What do you think about the overall topics of the sessions and how they progress over the 6 sessions?
   i) What do you think are the most impactful parts of PREPARED? *(Probe for enough details so that it's clear which sessions and/or skills they are referring to.)*
   ii) Why? *(Probe to understand how reasons are related to feasibility, acceptability, understandability, relevance/meaningfulness/helpfulness)*

c) What are parts of the PREPARED program that you didn't like as much? *(Probe for enough details so that it's clear which sessions and/or skills they are referring to.)*
   i) Why?
      (1) *Probe to understand how reasons are related to feasibility, acceptability, understandability, relevance/meaningfulness/helpfulness*

d) Do you think any important topics were missing?

e) How, if at all, do you think the youth in your group benefited from the program?
   i) *In terms of benefits, how would you compare it to other programs you have been exposed to in your community? Does anything about PREPARED stand out as unique?*

f) PREPARED was designed to help support youth well-being and healthy relationship development. We hope it prevents mental health problems and violence in dating relationships. Do you think we achieved that? Why or why not?

g) What was the more important part of PREPARED from your opinion?

h) I'll give you a minute to think about this next question. I'm wondering what was your most rewarding moment?

4) PREPARED Implementation

a) Now I'd like to ask you some practical questions about the program.
   i) What did you think about the facilitator training process?
      (1) Positive parts / negative?
      (2) How could it be improved for new facilitators in the future?
      (3) Is there anything you wish you could have been more involved in? Or learned more about?
   ii) How did you feel about delivering PREPARED at the school? In the community?

b) What did you think about the length of the program?
   i) What about the length of each session?

c) What did you think about the manual?
   i) What did you think about the written portions that you read from? Do you have any suggestions?
   ii) What did you think about other intervention materials?

d) There were many different types of activities in PREPARED: group discussions, activities, games. What were your favorite and least favorite <u>types</u> of activities (not necessarily specific ones)?
   i) Why? Do you have any ideas for changing the types that you did not like?

e) We know PREPARED was a big time commitment. Is there anything you can think of that could be shaved off, or cut, to reduce commitment required?

5) Closing and Thank you:

a) I think we are nearing the end of our time together. Before we close, *I wanted to take the opportunity to ask if there is anything you think I missed asking? Is there anything else*

you want to share about your role as a facilitator, your collaboration with the Duke team, or about the program itself?

b) Thank you so much for being here, I so appreciate the time you have taken to share your experience with us.

Youth Endline Focus Group Discussion
Interview Guide

[English]

1) **General**
   a) What was PREPARED like for you? I would love to hear both what you liked and what you did not like.
   b) *Dislike probe*: Can you think of anything that would have made that better?
   c) Were there things you thought were interesting or fun or helpful? How about things that were confusing or boring or not very useful? We want you to be totally honest.

2) **STORIES OF CHANGE:** Can you please give me one or two specific examples about how things were before you participated in PREPARED and how things are now? *[explanation probe if needed: It helps to hear about how these changes played out in real life. Can you think of something that happened before PREPARED - and then something that happened afterwards - that shows how things changed?]*
   a) What do you think caused these changes to happen?
      i) Which parts of PREPARED do you think might have led to this change?
      ii) What did you start doing differently that made the difference?
   b) ***If No Change or Worsening Probe****: PREPARED was designed to help support youth well-being and healthy relationships, but maybe that didn't happen for you. That is so important for us to learn about. Did things change for you? Or did anything get worse for you?*
   c) What are *other* changes that you think are related to being part of PREPARED? Things that we haven't talked about yet. *(Probe for examples and specific stories.)*
      i) *Allow for open-ended responses, but if needed you can also probe about changes to how they deal with stress, how they cope, how they think about dating relationships.*
      ii) Probes for any new change described:
         (1) STORIES OF CHANGE: Can you please give me a specific example, maybe about how things were before and how things are now?
         (2) CONTENT: Which parts of PREPARED do you think led to this change?
   d) Thank you. I have a couple of specific questions about other types of changes you may or may not have experienced.
      i) Have you noticed any changes in yourself?
         (1) Any changes in how you cope with negative things (events, thoughts, or feelings)?
         (2) Any changes in how you are in your job or school?

ii) Have you noticed any other changes in peer friendships?
iii) Have you noticed any other changes in boyfriend / girlfriend relationships?
iv) How about changes in relationships with your parents / caregivers?
v) In relationships with others outside of your friends and family such as changes at school with your teachers?
  (a) Not all changes are positive. Have you noticed any negative changes? *(only if they have only mentioned positive)*

3) **<u>Overall Skills Use and Content Usefulness</u>**
  a) Overall, which parts of PREPARED are you using in your daily life?
    i) Probes (only if needed to clarify/build on response):
      (1) What skills are you using from PREPARED?
        (a) *If having difficulty: By skills, I'm thinking like the Mountains and Valleys, Walk it out, Talk it out, Check it out, Map it Out, CLEAR skills, WORK it out skills, SAFE skills, etc.*
        (b) Why do you think you are still using those?
        (c) How are you still using those? & How are they helpful? *(do not ask if already explained fully in description of changes.)*
  b) What things from PREPARED are you not using in your daily life?
  c) What are any things that are keeping you from using skills that you learned?
  d) What is the more important or most helpful skill you learned? Why?

4) **<u>Engagement & Satisfaction</u>**
  a) What did you think of the program materials?
    i) Were any parts confusing?
    ii) What would make them better?
  b) What did you think about the length of the program?
    i) What about the length of each session?
  c) What did you think about how PREPARED was facilitated?
    i) What do you think of your facilitators?
    ii) Did you like meeting in groups?
      (1) What did you think about the parts when boys and girls were separate? What about the time all together?
    iii) What did you think about having the program at school (if in school group)? What did you think about having the program in the community? (if any community group)
  d) What was it that motivated you to come to PREPARED sessions? (Why did you want to come to them?)
  e) What made it difficult to come to sessions and/or participate in sessions?
    i) For barriers, what do you think could have improved or prevented this?
  f) Did you get out of PREPARED what you hoped to get out of it?
    i) Which parts did meet your expectations? Which parts did not?
  g) How would you describe PREPARED to other youth? Would you encourage them to join? ***[can skip if running out of time.]***
    i) Why or why not?

5) **Closing and thank you:**

a) We are nearing the end of our time together. Before we close, do you have any other thoughts about PREPARED, ideas for how it could be improved, or anything else you want to share?

Supplemental Material D

Excluded measures due to low reliability.

Emotion Regulation. Emotion regulation was assessed with 21 items from the Children's Emotion Management Scales (CEMS), including 10 items from the Children's Sadness Management Scale (CSMS) and 11 from the Children's Anger Management Scale (CAMS; Zeman et al., 2001). Both scales are scored to create inhibition, dysregulation, and coping subscales. Participants responded on a 3-point scale: "hardly ever" (1); "sometimes" (2); "often" (3). The Cronbach alphas across subscales ranged from 0.24 to 0.67, with four of the six <0.40. Given the lack of reliability, results of these scales are not reported.

Coping. Adolescent coping responses to stressors were assessed using 8 items from Brief COPE (Carver, 1997). Two-item subscales were calculated to measure four domains: active coping (α=0.57), planning-focused coping (α=0.52), religious coping (α=0.05), and substance-use as coping (α=0.71). Participants responded to items using a 4 point scale from "I haven't been doing this at all" (1) to "I've been doing this a lot" (4). Responses were averaged, with higher subscale scores indicating greater engagement with that coping style. Given the lack of reliability, results of these scales are not reported.

Supplemental Material E

Primary Intervention Outcomes by Implementation Setting

Primary Outcomes	Possible Range	Valence	Pre-Mean	Post-Mean	Hedge's g	95% CI
**In School (*N*=24)**						
Well-Being	1-5	+	21.95 (5.43)	3.84 (0.76)	0.81	0.07, 1.54
Depressive Symptoms	0-27	-	7.23 (4.06)	5.23 (4.92)	-0.43	-1.03, 0.18
Psychological Distress	0-24	-	9.14 (4.14)	7.64 (4.02)	-0.35	-0.97, 0.26
Perceived Social Support	7-84	+	58.27 (13.17)	67.05 (10.00)	0.72	0.10, 1.34
Violence Attitudes (Disagreement)	1-3	+	2.27 (0.49)	2.42 (0.56)	0.28	-0.25, 0.81
Positive Sexuality	1-7	+	4.90 (1.40)	5.75 (1.10)	0.66	-0.09, 1.41
**Community (*N*=22)**						
Well-Being	1-5	+	24.67 (4.28)	26.91 (6.41)	0.36	-0.25, 0.97
Depressive Symptoms	0-27	-	9.33 (5.68)	7.92 (5.32)	-0.25	-0.88, 0.38
Psychological Distress	0-24	-	9.75 (3.86)	8.54 (3.83)	-0.30	-0.81, 0.20
Perceived Social Support	7-84	+	59.93 (12.05)	66.71 (12.60)	0.53	-0.03, 1.10
Violence Attitudes (Disagreement)	1-3	+	2.29 (0.62)	2.37 (0.61)	0.12	-0.47, 0.71
Positive Sexuality	1-7	+	5.21 (1.01)	6.09 (0.83)	0.91	0.26, 1.57

Supplemental Material F

Primary Outcomes and Relationship Outcomes by Implementation Sex

Primary Outcomes	Possible Range	Valence	Pre-Mean	Post-Mean	Hedge's g	95% CI
**Female Participants (*N*=24)**						
Well-Being	1-5	+	22.50 (5.0)	27.33 (4.70)	0.96	0.28, 1.65
Depressive Symptoms	0-27	-	10.00 (4.93)	6.38 (5.10)	-0.70	-1.24, -0.15
Psychological Distress	0-24	-	10.08 (4.48)	7.96 (4.31)	-0.47	-1.08, 0.15
Perceived Social Support	7-84	+	57.76 (13.67)	67.71 (12.53)	0.73	0.14, 1.33
Violence Attitudes (Disagreement)	1-3	-	2.46 (0.48)	2.67 (0.39)	0.47	-0.24, 1.42
Positive Sexuality	1-7	+	5.29 (1.12)	6.02 (0.96)	0.67	0.08, 1.26
**Male Participants (*N*=22)**						
Well-Being	1-5	+	24.32 (4.94)	25.73 (5.95)	0.25	-0.36, 0.86
Depressive Symptoms	0-27	-	6.50 (5.58)	6.91 (5.53)	0.08	-0.56, 0.72
Psychological Distress	0-24	-	8.77 (3.28)	8.27 (3.51)	-0.14	-0.76, 0.48
Perceived Social Support	7-84	+	60.64 (11.18)	65.95 (10.01)	0.48	-0.27, 1.24
Violence Attitudes (Disagreement)	1-3	-	2.08 (0.58)	2.09 (0.61)	0.02	-0.51, 0.54
Positive Sexuality	1-7	+	4.18 (1.27)	5.83 (0.99)	0.86	0.09, 1.64
**Relationship Outcomes**	**Possible Range**	**Valence**	**Pre-Mean**	**Post-Mean**	**Hedge's g**	**95% CI**
**Female Participants (*N*=10)**						
Couples' Communication	1-6	+	5.24 (0.65)	5.77 (0.46)	0.87	-0.29, 2.02
Decision Making Power (Shared)	0-1	+	0.30 (0.19)	0.44 (0.17)	0.70	0.04, 1.37
Dehumanization of Self by Partner	1-7	-	2.37 (1.77)	1.40 (1.04)	-0.44	-1.24, 0.37
Dehumanization of Partner	1-7	-	2.30 (1.49)	1.67 (1.12)	-0.62	-1.67, 0.43
**Male Participants (*N*=10)**						
Couples' Communication	1-6	+	4.79 (1.29)	5.19 (0.69)	0.35	-0.55, 1.26
Decision Making Power (Shared)	0-1	+	0.41 (0.35)	0.54 (0.25)	0.38	-0.33, 1.08
Dehumanization of Self by Partner	1-7	-	3.33 (1.89)	2.70 (1.95)	-0.35	-1.26, 0.56
Dehumanization of Partner	1-7	-	3.03 (1.36)	2.47 (1.60)	-0.30	-1.25, 0.66

Supplemental Material G

**Sexual Risk Behaviors (*N*=21)**	**Pre *N(%)***	**Post *N(%)***
I have more than one sexual partner currently.	8(38)	3(20)
I use a condom.	11(52)	13(53)
I get paid or *my partner(s)* buys me things when I engage in sexual activity.	2(1)	0
I pay for sexual activity through money or buying *my partner(s)* things.	2(1)	1(0.5)
I have been tested for HIV or other sexually transmitted infections (STIs).	13(57)	16(76)
